# E3 ligase adaptor FBXO7 contributes to ubiquitination and proteasomal degradation of SIRT7 and promotes cell death in response to hydrogen peroxide

**DOI:** 10.1016/j.jbc.2023.102909

**Published:** 2023-01-13

**Authors:** Su Hyoun Lee, Yun Ju Lee, Sungyeon Jung, Kwang Chul Chung

**Affiliations:** Department of Systems Biology, College of Life Science and Biotechnology, Yonsei University, Seoul, Korea

**Keywords:** Parkinson’s disease, FBXO7, SIRT7, ubiquitin E3 ligase, aging, proteasome degradation, hydrogen peroxide, 6-OHDA, 6-hydroxydopamine, CUL1, Cullin-1, DMEM, Dulbecco’s modified Eagle’s medium, FBP, F-box protein, FBS, fetal bovine serum, FBXO7, F-box-only protein 7, H_2_O_2_, hydrogen peroxide, HEK293, human embryonic kidney 293 cell line, HRP, horseradish peroxidase, LDH, lactate dehydrogenase, MPP^+^, 1-methyl-4-phenylpyridinium, PD, Parkinson’s disease, PINK1, PTEN-induced kinase 1, SCF, SKP1–Cullin–1–F-box, SIRT7, sirtuin 7, TBST, Tris-buffered saline with Tween-20, Ubl, ubiquitin-like, UPS, ubiquitin proteasome system

## Abstract

Parkinson’s disease (PD) is a degenerative disorder of the central nervous system that affects 1% of the population over the age of 60. Although aging is one of the main risk factors for PD, the pathogenic mechanism of this disease remains unclear. Mutations in the F-box-only protein 7 (FBXO7) gene have been previously found to cause early onset autosomal recessive familial PD. FBXO7 is an adaptor protein in the SKP1–Cullin–1–F-box (SCF) E3 ligase complex that facilitates the ubiquitination of substrates. Sirtuin 7 (SIRT7) is an NAD^+^-dependent histone deacetylase that regulates aging and stress responses. In this study, we identified FBXO7 as a novel E3 ligase for SIRT7 that negatively regulates intracellular SIRT7 levels through SCF-dependent Lys-48-linked polyubiquitination and proteasomal degradation. Consequently, we show that FBXO7 promoted the blockade of SIRT7 deacetylase activity, causing an increase in acetylated histone 3 levels at the Lys-18 and Lys-36 residues and the repression of downstream *RPS20* gene transcription. Moreover, we demonstrate that treatment with hydrogen peroxide triggered the FBXO7-mediated degradation of SIRT7, leading to mammalian cell death. In particular, the PD-linked FBXO7-R498X mutant, which reduced SCF-dependent E3 ligase activity, did not affect the stability of SIRT7. Collectively, these findings suggest that FBXO7 negatively regulates SIRT7 stability and may suppress the cytoprotective effects of SIRT7 during hydrogen peroxide–induced mammalian cell death.

Parkinson’s disease (PD) is a common neurodegenerative disease caused by the loss of dopaminergic neurons in the substantia nigra of the midbrain (1). Multiple factors, including abnormal protein aggregation, oxidative damage, brain inflammation, and mitochondrial dysfunction, have been suggested to play critical roles in PD pathogenesis (2, 3). Based on the previous finding that PD typically manifests after the age of 60 years and aging-related changes are closely related to neuronal cell death, aging has also been suggested as one of the most important risk factors for the development of PD (3, 4, 5). However, the correlation between aging and PD and the underlying mechanisms remain unclear. Although most cases of PD are sporadic, 5 to 10% of cases develop at a much younger age, which are caused by mutations in one of several familial genes, including α-synuclein, PTEN-induced kinase 1 (*PINK1*), parkin, *LRRK2*, *DJ-1*, *ATP13A2*, and *FBXO7* (6, 7).

FBXO7 is an F-box protein (FBP) containing an F-box domain and acts as a substrate recognition adaptor protein of Skp1–Cullin-1 (CUL1)–F-Box (SCF) E3 ligases complexes (8). FBXO7 has five domains (9), including the N-terminal ubiquitin-like (Ubl) domain, Cdk6 domain, FP domain, F-box domain, and C-terminal proline-rich region. To date, three missense or nonsense point mutations of FBXO7 (T22M, R378G, and R498X) have been identified to be associated with familial forms of PD (10, 11). Unlike most other FBPs, FBXO7 has both SCF-independent and SCF-dependent functions (8). In SCF-independent roles, FBXO7 regulates proteasome activity, cell cycle progression, and mitophagy (12, 13, 14). In addition, we recently revealed that FBXO7 interacts with caspase-8 to cause its activation, leading to degradation of the aging-related transcription factor FOXO4 in mammalian cells (15). In a standard SCF-dependent manner, FBXO7 interacts with several substrates and mediates their ubiquitination, thus playing a role in various cellular processes, including cell cycle regulation and mitophagy (16, 17, 18, 19, 20). For example, the SCF–FBXO7 interaction mediates the ubiquitination and degradation of PINK1 (21). Small-molecule inhibitors of FBXO7 that interfere with the FBXO7–PINK1 interaction reduce mitochondrial injury and inflammation, leading to neuroprotection in primary cortical neurons, human neuroblastoma cells, and PD patient–derived cells (21).

Silent information regulator 2 is an NAD^+^-dependent protein deacetylase and extends the life span of yeast (22). Sirtuins are mammalian homologs of silent information regulator 2, which share a conserved NAD^+^-dependent catalytic domain, localize to different subcellular compartments, and regulate various physiological processes, including cell cycle progression, proliferation, apoptosis, metabolism, genomic stability, and aging (23). Therefore, dysregulation of proper sirtuin functions is implicated in the pathogenesis of many human diseases, such as cancer, type II diabetes, and neurodegenerative diseases. Among the seven mammalian sirtuins, SIRT7 predominantly localizes to the nucleus and is involved in various cellular processes, including metabolism, ribosome biogenesis, and stress resistance (24, 25). Many studies have revealed that sirtuins affect several life extension pathways as well as brain function, which could offer new targets in the treatment of neurodegenerative diseases, including Alzheimer’s disease and PD (26, 27). For example, SIRT1 was found to protect human neuroblastoma SH-SY5Y cells by downregulating the expression of NF-κB and cleaved poly(ADP-ribose) polymerase 1 and by reducing the formation of phospho-α-Syn aggregates, whereas SIRT2 promoted toxic protein aggregation by deacetylating α-Syn and α-tubulin, thereby exacerbating the pathogenesis of PD (28). Furthermore, SIRT3 was reported to protect cells against 1-methyl-4-phenylpyridinium^+^ (MPP^+^)-induced neuronal injury (29). However, little is known about the functionality of SIRT7 with respect to cell viability and its implication in the progression of PD.

Based on accumulating evidence that several FBPs and sirtuins are functionally linked, in this study, we further examined whether and how mammalian sirtuins and FBXO7 are closely associated. We here show that FBXO7 specifically binds to SIRT7. In addition, we found that FBXO7 acts as a novel ubiquitin E3 ligase that targets SIRT7, promoting its ubiquitination and subsequent SCF-dependent proteasomal degradation. Furthermore, FBXO7-mediated degradation of SIRT7 was observed during hydrogen peroxide (H_2_O_2_)–induced cell death in SH-SY5Y cells, which considerably promoted the cytotoxicity. Taken together, our study implies that the functional relationship between FBXO7 and cytoprotective SIRT7 in a reverse manner may play a role in H_2_O_2_-induced cell death and could contribute to the pathogenesis of PD.

## Results

### FBXO7 interacts with and affects SIRT7 in mammalian cells

**Figure 1:**
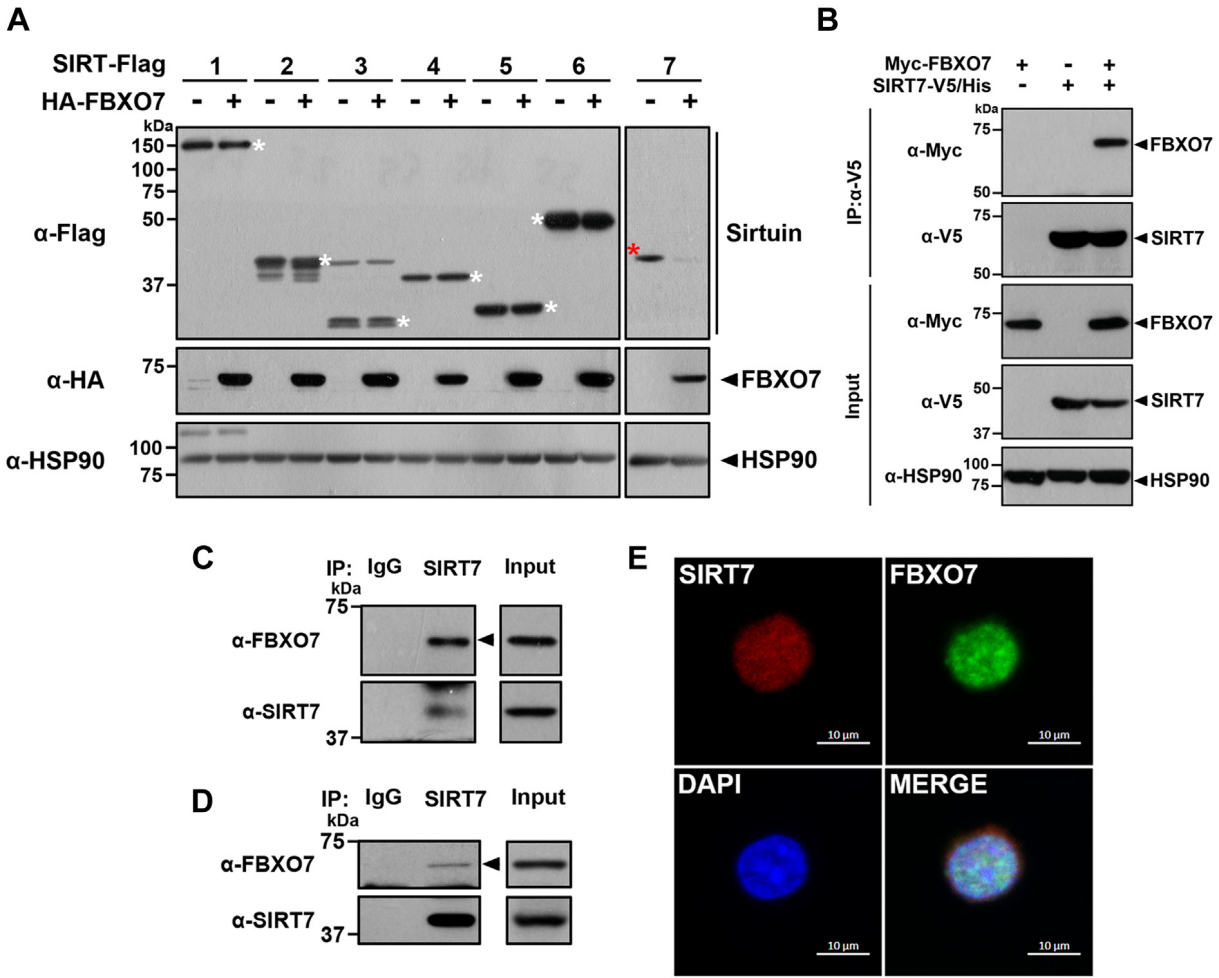
**FBXO7 interacts with SIRT7.***A*, HEK293 cells were transfected for 24 h with a plasmid encoding FLAG-tagged SIRT1, 2, 3, 4, 5, 6, and7 and/or HA-FBXO7. Cell lysates were immunoblotted with anti-FLAG or anti-HA antibodies. *B*, HEK293 cells were transfected with a plasmid encoding Myc-FBXO7 and/or SIRT7-V5/His. Cell lysates were immunoprecipitated using an anti-V5 antibody, followed by immunoblotting with the indicated antibodies. HSP90 served as a loading control. *C*, HEK293 cell lysates were immunoprecipitated using either anti-SIRT7 antibody or preimmune IgG (control), followed by immunoblotting with the indicated antibodies. *D*, mouse whole-brain lysates were immunoprecipitated using anti-SIRT7 IgG, followed by immunoblotting with the indicated antibodies. *E*, SH-SY5Y cells were treated for 6 h with 20 μM MG132 and then fixed, permeabilized, and immunostained. Representative confocal images of cells immunostained for endogenous SIRT7 (*red*) and endogenous FBXO7 (*green*) are shown. Nuclei were counterstained with DAPI (*blue*). The scale bars represent 10 μm. DAPI, 4′,6-diamidino-2-phenylindole; FBXO7, F-box-only protein 7; HA, hemagglutinin; HEK293, human embryonic kidney 293 cell line; HSP90, heat shock protein 90; IgG, immunoglobulin G; SIRT7, sirtuin 7.

Based on our previous finding that FBXO7 exacerbates 6-hydroxydopamine (6-OHDA)-induced neuronal cell death by interacting with aging-related FOXO4 and promoting its degradation (15), we speculated that FBXO7 may also be physically and/or functionally related to sirtuins, another family of crucial aging-related genes. To investigate whether this hypothesis is valid, we first checked whether FBXO7 affects the levels of the seven sirtuins when they were coexpressed. When human embryonic kidney 293 (HEK293) cells were transiently transfected with a plasmid encoding one of the seven sirtuins with a FLAG tag at the C terminus alone or together with HA-FBXO7, ectopically expressed FBXO7 effectively reduced the level of SIRT7, whereas the other six sirtuin levels were unaffected (Fig. 1*A*). Unlike other sirtuins, the putative role of SIRT7 in cell viability and its functional regulation have not been elucidated; therefore, we further assessed the link between FBXO7 and SIRT7 and particularly focused on whether SIRT7 may act as a new target of FBXO7.

We confirmed that ectopically expressed SIRT7 interacted with FBXO7 in HEK293 cells (Fig. 1*B*). The biochemical interaction between endogenous FBXO7 and SIRT7 was also confirmed in HEK293 cells (Fig. 1*C*). In addition, the interaction between endogenous FBXO7 and SIRT7 was verified in mouse whole-brain lysates (Fig. 1*D*). These results suggested that the observed FBXO7–SIRT7 interaction was not an artifact of DNA transfection in immortalized cancer cells but rather represents a specific interaction in mammalian cells. Subsequently, we examined whether the two proteins colocalized in human neuroblastoma SH-SY5Y cells. Since endogenous SIRT7 is quickly degraded, the cells were treated with MG132, and immunohistochemical analysis of SH-SY5Y cells revealed that endogenous FBXO7 and SIRT7 colocalized mainly in the nucleus (Fig. 1*E*).

To clarify the FBXO7 domain(s) responsible for the SIRT7 interaction, several FLAG-tagged FBXO7-deletion mutants were generated (Fig. S1*A*). Each of these mutants lacked one of the following wildtype protein domains: the Ubl domain (▵U), F-box domain (▵F), C-terminal region of amino acids 376 to 522 (▵C), or N-terminal region of amino acids 1 to 333 (▵N). HEK293 cells were then transfected with the plasmid encoding FLAG-tagged wildtype FBXO7 alone, together with wildtype SIRT7-V5/His or one of the deletion mutants. After coimmunoprecipitation analysis of cell lysates with an anti-V5 antibody, immunoblotting analysis revealed that SIRT7 bound to FBXO7-▵U, FBXO7-▵F, and FBXO7-▵C, as well as its binding to full-length FBXO7. However, this interaction was not observed for FBXO7-▵N (Fig. S1*B*).

Taken together, these results suggested that FBXO7 binds to SIRT7 and affects its expression level in mammalian cells and that the region spanning amino acid residues 92 to 333 of FBXO7, including the FP domain, is important for SIRT7 binding.

### FBXO7 promoted the degradation of SIRT7

**Figure 2:**
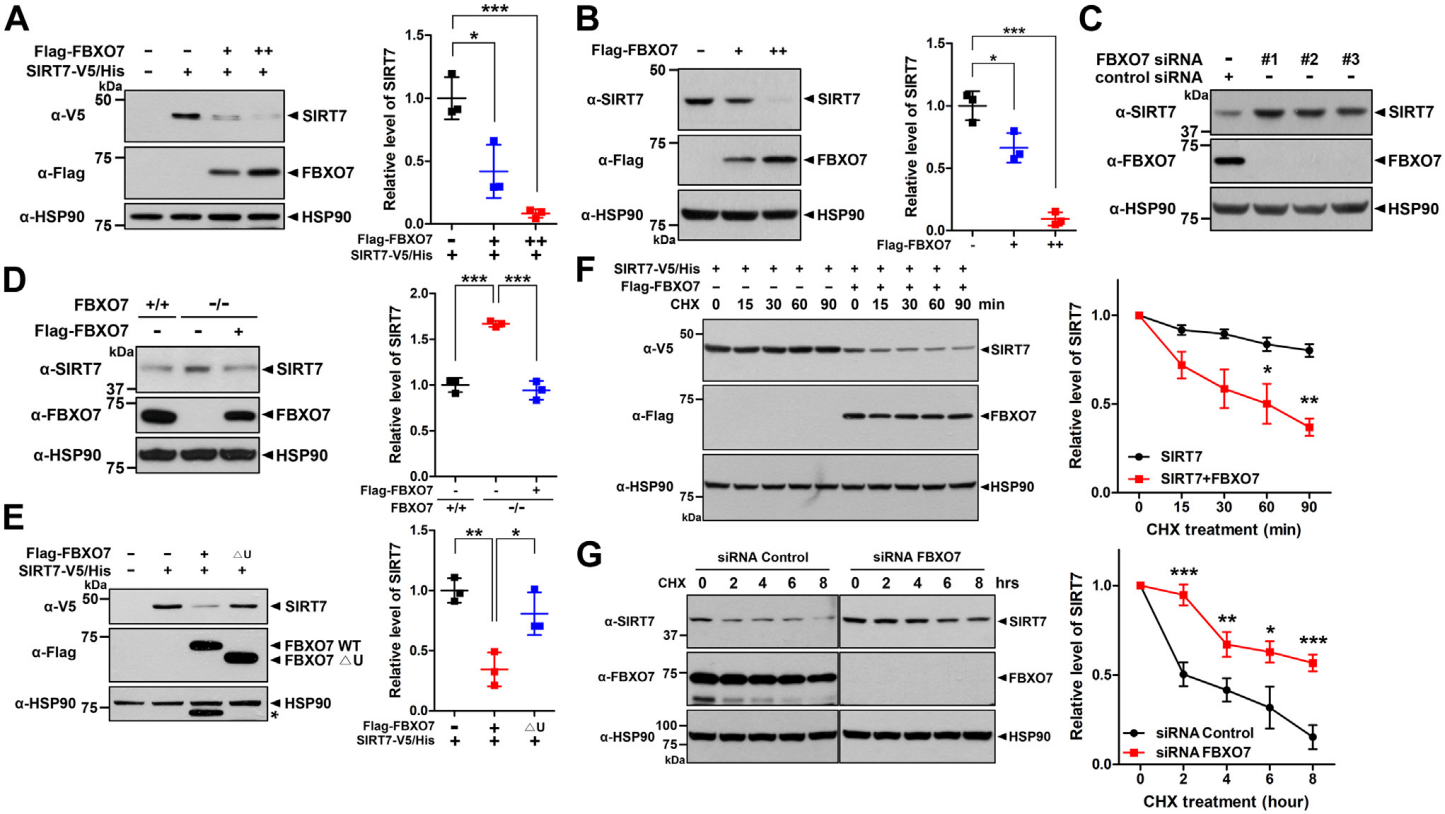
**FBXO7 promotes the degradation of SIRT7.***A*, HEK293 cells were transfected for 24 h with the plasmids encoding wildtype SIRT7-V5/His alone or together with increasing amounts of plasmid encoding FLAG-FBXO7. Cell lysates were immunoblotted with anti-V5 or anti-FLAG antibodies. Relative SIRT7 levels were quantified, and the results are presented as the mean ± SD of three independent experiments (∗∗∗*p* ≤ 0.0001; ∗*p* ≤ 0.05). *B*, HEK293 cells were transfected for 24 h with increasing amounts of a plasmid encoding FLAG-FBXO7-WT. Cell lysates were immunoblotted and screened with anti-SIRT7 or anti-FLAG antibodies. Related levels of SIRT7 were quantified, and results are presented as the mean ± SD of three independent experiments (∗∗∗*p* ≤ 0.0001; ∗*p* ≤ 0.05). *C*, HEK293 cells were transfected for 48 h with control-siRNA or *FBXO7*-siRNA. Cell lysates were immunoblotted with anti-SIRT7 or anti-FBXO7 antibodies. *D*, FBXO7^+/+^ and FBXO7^−/−^ HAP1 cells were transfected for 24 h with a plasmid encoding FLAG-FBXO7. Cell lysates were immunoblotted with anti-SIRT7 or anti-FBXO7 antibodies. Relative SIRT7 levels were quantified and presented as the mean ± SD of three independent experiments (∗∗∗*p* ≤ 0.0001). *E*, HEK293 cells were transfected for 24 h with a plasmid encoding wildtype SIRT7-V5/His alone or together with either FLAG-FBXO7-WT or FLAG-FBXO7-▵U. Cell lysates were immunoblotted with the indicated antibodies. Relative SIRT7 levels were quantified, and the results are presented as the mean ± SD of three independent experiments (∗∗*p* ≤ 0.001; ∗*p* ≤ 0.05). *Asterisks* indicate nonspecific bands. *F*, HEK293 cells were transfected for 24 h with plasmids encoding wildtype SIRT7-V5/His and/or FLAG-FBXO7-WT. Cells were treated for the indicated times with 25 μg/ml cycloheximide, and cell lysates were immunoblotted with the indicated antibodies. Relative SIRT7 levels were quantified, and the results are presented as the mean ± SD of three independent experiments (∗∗*p* ≤ 0.001; ∗*p* ≤ 0.05). *G*, HEK293 cells were transfected for 48 h with control-siRNA or *FBXO7*-siRNA. Cells were treated for the indicated times with 25 μg/ml cycloheximide, and cell lysates were immunoblotted with the indicated antibodies. Relative levels of SIRT7 were quantified, and the results are presented as the mean ± SD of three independent experiments (∗∗∗*p* ≤ 0.0001; ∗∗*p* ≤ 0.001; and ∗*p* ≤ 0.05). HSP90 served as a loading control. FBXO7, F-box-only protein 7; HEK293, human embryonic kidney 293 cell line; HSP90, heat shock protein 90; SIRT7, sirtuin 7.

To gain further insight into the link between FBXO7 and SIRT7, we examined whether FBXO7, as a component of SCF E3 ligase, negatively regulates the level of SIRT7. Similar to the pattern shown in Figure 1, *A* and *B*, exogenous and endogenous SIRT7 levels were significantly reduced in the presence of FBXO7 in a dose-dependent manner (Fig. 2, *A* and *B*). In contrast, siRNA-mediated *FBXO7* knockdown in HEK293 cells significantly increased the endogenous SIRT7 levels (Fig. 2*C*). In addition, endogenous SIRT7 levels were significantly increased in *FBXO7*-knockout HAP1 cells (Fig. 2*D*). Moreover, reintroduction of wildtype FBXO7 into *FBXO7*-knockout cells reduced endogenous SIRT7 levels (Fig. 2*D*).

To determine whether the catalytic activity of FBXO7 is required for SIRT7 downregulation, HEK293 cells were transfected for 24 h with plasmids encoding SIRT7-V5/His alone or together with FLAG-FBXO7-WT or FLAG-FBXO7-▵U, which lacks the Ubl domain and thus acts as a catalytically inactive mutant in SCF. The FBXO7-▵U mutant had no effect on SIRT7 levels, in contrast to the effect of FBXO7-WT (Fig. 2*E*). These results suggested that the catalytic activity of FBXO7 is critical for SIRT7 reduction.

Next, we assessed whether FBXO7 regulates the stability of SIRT7 by measuring the SIRT7 half-life. HEK293 cells were transfected with plasmids encoding V5/His-tagged SIRT7, either alone or in combination with FLAG-FBXO7, and treated with 25 μM cycloheximide for various times. Immunoblot analysis revealed that the half-life of SIRT7 was significantly decreased by FBXO7 (Fig. 2*F*). In contrast, the half-life of endogenous SIRT7 was stabilized by siRNA-mediated *FBXO7* knockdown (Fig. 2*G*). Taken together, these results suggested that FBXO7 promoted the degradation of SIRT7.

### FBXO7 decreased SIRT7 stability through SCF complex–dependent proteasomal degradation

**Figure 3:**
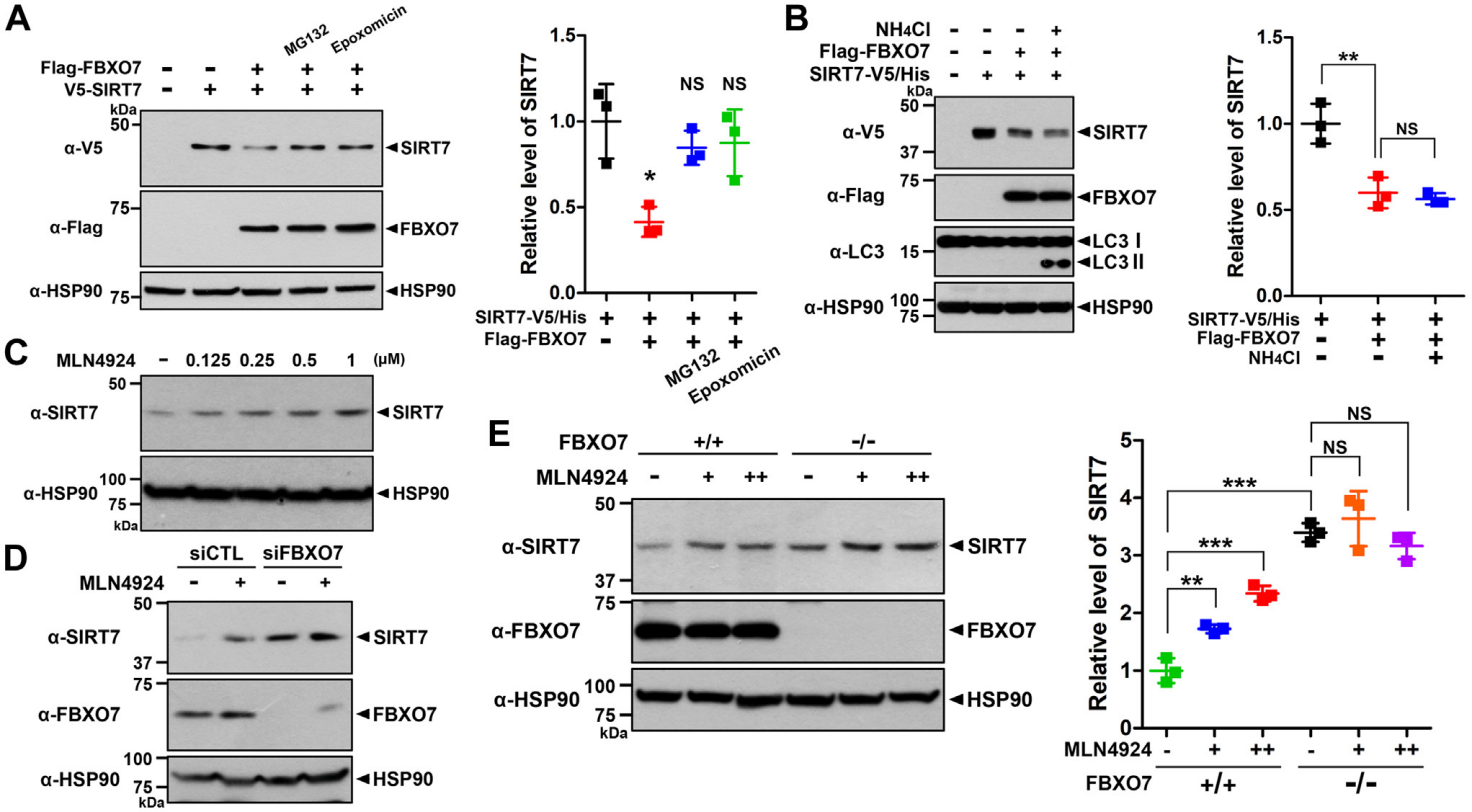
**FBXO7-mediated decrease of SIRT7 occurs through SCF complex–dependent proteasome degradation.***A*, HEK293 cells were transfected for 24 h with plasmids encoding SIRT7-V5/His and/or FLAG-FBXO7. The cells were then treated for an additional 6 h with vehicle (−), 20 μM MG132, or 0.5 μM epoxomicin, as indicated. Relative SIRT7 levels were quantified, and the results are presented as the mean ± SD of three independent experiments (∗*p* ≤ 0.05; NS, not significant). *B*, HEK293 cells were transfected for 24 h with plasmids encoding SIRT7-V5/His and/or FLAG-FBXO7 and treated for an additional 6 h with vehicle (−) or 10 mM NH_4_Cl. Relative SIRT7 levels were quantified, and the results are presented as the mean ± SD of three independent experiments (∗∗*p* ≤ 0.001; NS). *C*, HEK293 cells were treated for 24 h with vehicle (−) or the indicated concentrations of MLN4924. Cell lysates were immunoblotted with the indicated antibodies. *D*, HEK293 cells were transfected for 48 h with control-siRNA or *FBXO7*-siRNA. The cells were then treated for an additional 24 h with vehicle (−), 1.5 μM MLN4924, and cell lysates were immunoblotted with the indicated antibodies. *E*, where specified, FBXO7^+/+^ and FBXO7^−/−^ HAP1 cells were treated with the vehicle (−) or MLN4924 (1 or 2 μM). Cell lysates were immunoblotted with the indicated antibodies. Relative SIRT7 levels were quantified, and the results are presented as the mean ± SD of three independent experiments (∗∗∗*p* ≤ 0.0001; ∗∗*p* ≤ 0.001; NS). HSP90 served as a loading control. FBXO7, F-box-only protein 7; HEK293, human embryonic kidney 293 cell line; HSP90, heat shock protein 90; SCF, SKP1–Cullin–1–F-box; SIRT7, sirtuin 7.

Next, we determined whether FBXO7-mediated SIRT7 degradation occurs through one of two intracellular proteolysis systems: the ubiquitin proteasome system (UPS) or the lysosome-involved autophagy pathway. HEK293 cells were transfected for 24 h with plasmid encoding SIRT7-V5/His or FLAG-FBXO7 alone or in combination and then further treated with either a proteasome inhibitor (MG132 or epoxomicin) or a lysosomal autophagy inhibitor (NH_4_Cl). As shown in Figure 3*A*, cells treated with the proteasome inhibitor exhibited restored SIRT7 levels. However, treatment with NH_4_Cl had no effect on the FBXO7-mediated reduction of SIRT7 levels (Fig. 3*B*), suggesting that the degradation of SIRT7 occurs through the UPS.

We then examined whether the proteolysis of SIRT7 by FBXO7 occurred through the SCF complex–dependent pathway using MLN4924, a commonly employed chemical inhibitor of SCF. Specifically, MLN4924 acts as an inhibitor of the NEDD8-activating enzyme, preventing the NEDDylation of cullin and thereby inactivating cullin-RING E3 ligase activity (30). When HEK293 cells were pretreated with MLN4924, endogenous SIRT7 accumulated in the presence of FBXO7 in a dose-dependent manner (Fig. 3*C*). To verify the involvement of the SCF complex in FBXO7-mediated SIRT7 degradation, HEK293 cells were transfected with either control siRNA or siRNA-*FBXO7* for 48 h, followed by treatment with vehicle or MLN4924. Consistent with the results shown in Figure 3*C*, MLN4924 treatment increased SIRT7 levels compared with those of cells transfected with control siRNA (Fig. 3*D*). However, this effect was not observed in cells transfected with *FBXO7*-siRNA (Fig. 3*D*). Likewise, the accumulation of SIRT7 was not seen in *FBXO7*-knockout cells (Fig. 3*E*). These results revealed that SIRT7 expression was reduced through SCF^FBXO7^-dependent proteasomal degradation.

### FBXO7 promoted Lys 48-linked polyubiquitination of SIRT7

**Figure 4:**
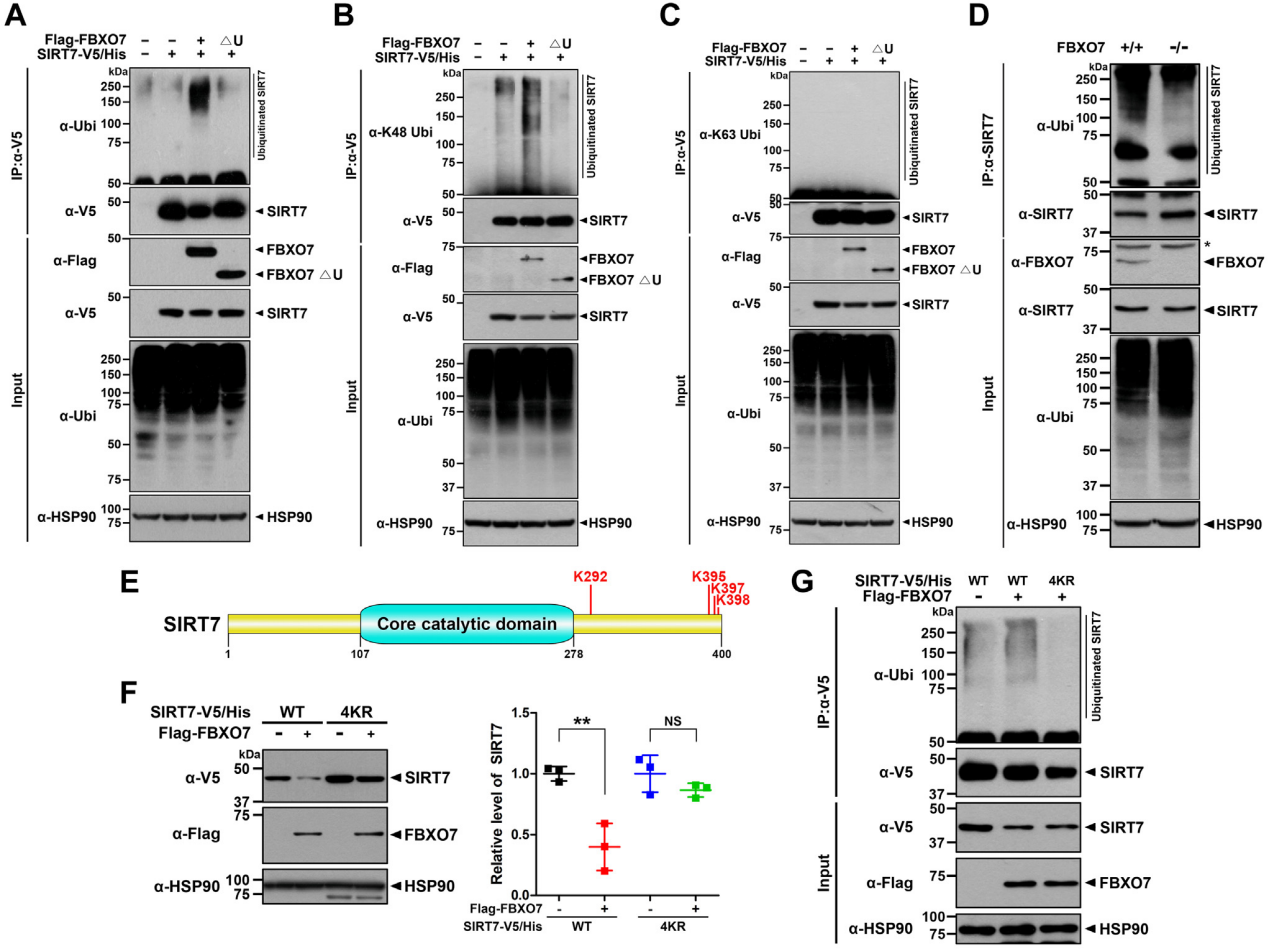
**FBXO7 promotes the K48-linked polyubiquitination of SIRT7.***A*–*C*, where indicated, HEK293 cells were transfected for 24 h with plasmids encoding SIRT7-V5/His alone or in combination with FLAG-FBXO7-WT or FLAG-FBXO7-▵U and treated for an additional 6 h with 20 μM MG132. Cell lysates were immunoprecipitated using anti-V5 antibody, followed by immunoblotting with the indicated antibodies. *D*, FBXO7^+/+^ and FBXO7^−/−^ HAP1 cells were treated for 6 h with 20 μM MG132. Cell lysates were immunoprecipitated using an anti-SIRT7 antibody, and the precipitates were immunoblotted with the indicated antibodies. *Asterisks* indicated nonspecific bands. *E*, schematic diagram of multiubiquitination sites on SIRT7 identified through ubiquitination assays. *F*, HEK293 cells were transfected for 24 h with plasmids encoding SIRT7-V5/His-WT, SIRT7-V5/His-4KR, and/or FLAG-FBXO7. Cell lysates were immunoblotted with the indicated antibodies. Relative levels of SIRT7 were quantified, and the results are presented as the mean ± SD of three independent experiments (∗∗*p* ≤ 0.001; NS, not significant). *G*, HEK293 cells were transfected for 24 h with plasmids encoding SIRT7-V5/His-WT, SIRT7-V5/His-4KR, and/or FLAG-FBXO7. The cells were then treated for an additional 6 h with 20 μM MG132. Cell lysates were immunoprecipitated using an anti-V5 antibody, followed by immunoblotting with the indicated antibodies. HSP90 served as a loading control. FBXO7, F-box-only protein 7; HEK293, human embryonic kidney 293 cell line; HSP90, heat shock protein 90; SIRT7, sirtuin 7.

As SIRT7 levels were significantly reduced through SCF^FBXO7^-dependent proteasome degradation (Fig. 3), we determined that it is highly probable that SIRT7 could be polyubiquitinated by FBXO7. To explore this possibility, HEK293 cells were transfected for 24 h with plasmids encoding SIRT7-V5/His alone or together with FLAG-FBXO7-WT or FLAG-FBXO7-▵U, followed by immunoprecipitation of the cell lysates with anti-V5 antibody. Immunoblotting of the samples with anti-FLAG antiserum revealed that wildtype FBXO7, but not FBXO7-▵U mutant, promotes the polyubiquitination of SIRT7 (Fig. 4*A*).

We then examined whether the FBXO7-mediated polyubiquitination of SIRT7 specifically targets either K48- or K63-linked chains. As expected, FBXO7-mediated SIRT7 polyubiquitination was significantly increased by proteasome-linked anti-K48-linked-ubiquitin antibody but not by anti-K63-linked-ubiquitin antibody (Fig. 4, *B* and *C*). In addition, SIRT7 polyubiquitination was reduced in *FBXO7*-knockout cells (Fig. 4*D*). These results demonstrated that FBXO7 directly mediates the polyubiquitination of SIRT7 by targeting K48-linked polyubiquitin chains.

We further examined whether the FBXO7-mediated polyubiquitination of SIRT7 was dependent on the SCF complex. When HEK293 cells were transfected with FLAG-FBXO7, the polyubiquitination of SIRT7 was markedly increased in cells transfected with control siRNA, whereas this increase was not observed in cells transfected with *CUL1*-siRNA, targeting a component of the SCF complex (Fig. S2). These results further suggested that FBXO7 promotes polyubiquitination of SIRT7 in an SCF complex–dependent manner.

Next, we attempted to identify the site(s) of SIRT7 that undergo FBXO7-meditated ubiquitination. A total of 21 lysine sites within SIRT7 were individually replaced with arginine one or two sites at a time, and we examined whether FBXO7-mediated SIRT7 ubiquitination could be still observed using those SIRT7 mutants. Among the 19 mutants, we found that SIRT7 mutants with substitutions at K292R, K395R, and K397/K398R displayed significantly reduced FBXO7-mediated ubiquitination (Fig. S3, *A* and *B*) compared with that of wildtype SIRT7. These four residues were then mutated to arginine (SIRT7-4KR), and the same experiments were performed (Fig. 4*E*). Compared with that of SIRT7-WT, the SIRT7-4KR mutant level was not considerably reduced by FBXO7 (Fig. 4*F*). In addition, FBXO7-mediated ubiquitination of the SIRT7-4KR mutant was markedly reduced compared with that of SIRT7-WT (Fig. 4*G*). These results suggested that FBXO7 mediates the polyubiquitination of SIRT7 by targeting at least four lysine sites of SIRT7: K292, K395, K397, and K398.

### FBXO7 increased the acetylated levels of H3K18 and H3K36 through SIRT7 degradation

**Figure 5:**
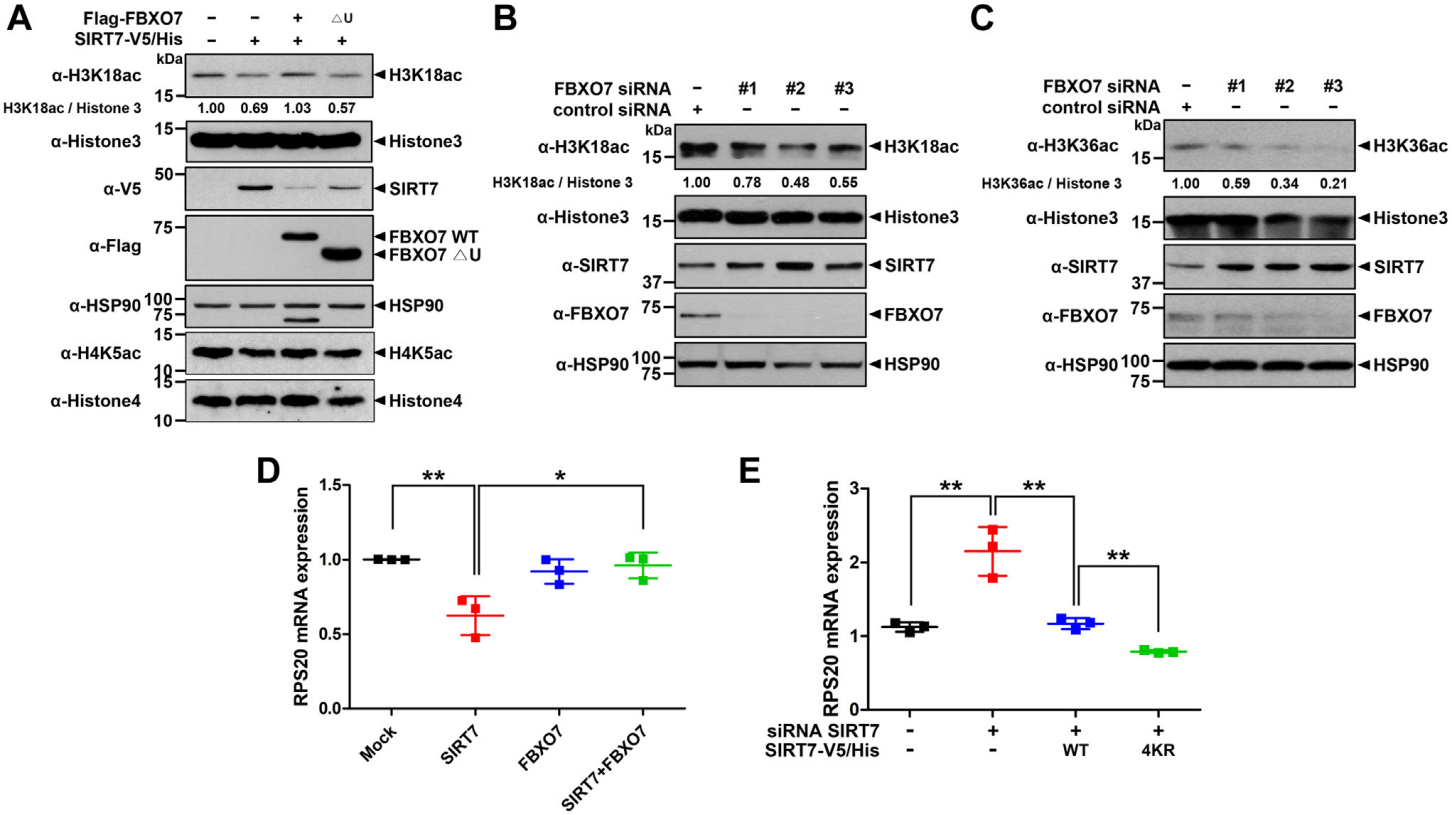
**FBXO7 promotes the acetylation of H3K18 and H3K36 through SIRT7 degradation.***A*, where specified, HEK293 cells were transfected for 24 h with plasmids encoding SIRT7-V5/His alone or together with either FLAG-FBXO7-WT or FLAG-FBXO7-▵U. Cell lysates were immunoblotted with the indicated antibodies. *B* and *C*, HEK293 cells were transfected for 48 h with control-siRNA, *FBXO7*-siRNA#1, *FBXO7*-siRNA#2, or *FBXO7*-siRNA#3. Cell lysates were immunoblotted with the indicated antibodies. The numbers at the *bottom* of the “H3K18ac” or “H3K36ac” panel indicate the quantitated values of fold induction measured using GelQuant.NET software. HSP90 served as a loading control. *D*, HEK293 cells were transfected for 24 h with plasmids encoding SIRT7-V5/His and/or FLAG-FBXO7. Total RNA was extracted and reverse-transcribed, and then the levels of *RPS20* mRNA were assessed using real-time PCR. Data are presented as the mean ± SD of three independent experiments (∗∗*p* ≤ 0.001; ∗*p* ≤ 0.05). *E*, HEK293 cells were transfected for 48 h with control-siRNA (CTL), *SIRT7*-siRNA, and a plasmid encoding SIRT7-V5/His WT or its 4KR mutant. Total RNA was extracted and reverse-transcribed. The levels of *RPS20* mRNA were assessed using real-time PCR. Data are presented as the mean ± SD of three independent experiments (∗∗*p* ≤ 0.001). FBXO7, F-box-only protein 7; HEK293, human embryonic kidney 293 cell line; HSP90, heat shock protein 90; SIRT7, sirtuin 7.

In addition to multiple histones, the deacetylase SIRT7 binds to DNA polymerase I, II, III, and several transcription factors, facilitating DNA transcription (31). In addition, SIRT7 has a specific activity to catalyze deacetylation of H3K18 and H3K36 (31, 32). Based on this report, we investigated whether FBXO7 controls the acetylation of H3K18 *via* SIRT7 degradation. To examine whether the FBXO7-mediated degradation of SIRT7 regulates the endogenous acetylated H3K18 level, HEK293 cells were transfected with plasmids encoding SIRT7-V5/His alone or in combination with FLAG-FBXO7-WT or FLAG-FBXO7-▵U. Immunoblot analysis of cell lysates using an anti-H3K18ac antibody revealed that acetylated H3K18 levels were decreased by SIRT7 alone (Fig. 5*A*). In contrast, FBXO7-WT, but not FBXO7-▵U, increased acetylated H3K18 levels *via* the reduction of SIRT7 (Fig. 5*A*). Moreover, among *FBXO7*-specific siRNA #1, #2, and #3, downregulation of acetylated H3K18 and upregulation of SIRT7 were observed most strongly in cells transfected with *FBXO7*-siRNA #2 (Fig. 5*B*). Likewise, acetylated H3K36 levels were also decreased by siRNA-mediated *FBXO7* knockdown (Fig. 5*C*).

Based on the report that SIRT7 induces the deacetylation of H3K18 at the promoter region of *RPS20*, resulting in the repression of its transcription (31), we further assessed and compared the changes in *RPS20* mRNA expression in the absence and presence of FBXO7. Real-time PCR analysis revealed that cells overexpressing exogenous SIRT7, which was used as a positive control, had approximately 40% lower *RPS20* mRNA expression levels than those of controls (Fig. 5*D*). By contrast, cells overexpressing exogenous FBXO7 showed no change in *RPS20* mRNA levels compared with those of mock-transfected cells (Fig. 5*D*). Furthermore, the cells coexpressing exogenous SIRT7 and FBXO7 showed significantly increased *RPS20* mRNA levels when compared with those of cells overexpressing SIRT7 alone (Fig. 5*D*).

To verify the effect of FBXO7-mediated polyubiquitination and the proteolysis of SIRT7 on *RPS20* transcription, we further examined the influence of SIRT7-4KR on *RPS20* mRNA expression. After endogenous SIRT7 expression was depleted using *SIRT7*-specific siRNA, either SIRT7-WT or SIRT7-4KR was reintroduced into *SIRT7*-depleted cell. Similar to the patterns shown in Figure 5*D*, real-time PCR analyses demonstrated that *RPS20* mRNA expression levels were significantly increased by siRNA-mediated *SIRT7* knockdown. In addition, *SIRT7*-knockdown cells reintroduced with SIRT7-4KR mutant displayed more strongly decreased *RPS20* mRNA expression compared with that of *SIRT7*-knockdown cells reintroduced with SIRT7-WT (Fig. 5*E*). These data indicated that endogenous FBXO7 might not affect the function of the SIRT7-4KR mutant.

Collectively, these results suggested that FBXO7 negatively regulates the deacetylase activity of SIRT7 *via* ubiquitination and degradation.

### H_2_O_2_ treatment reduced SIRT7 levels *via* FBXO7-mediated degradation in SH-SY5Y cells

**Figure 6:**
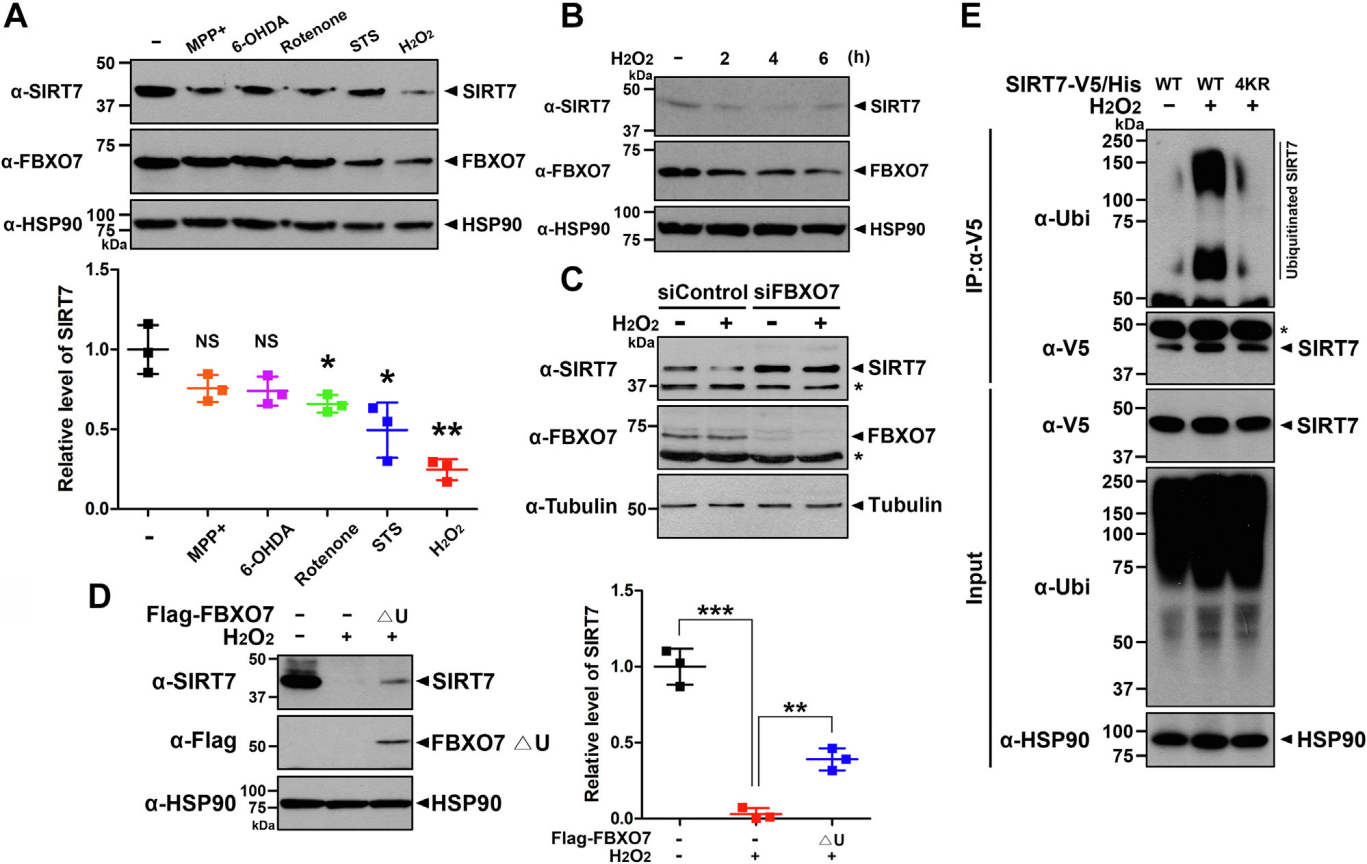
**Hydrogen peroxide (H**_**2**_**O**_**2**_**) treatment reduces the SIRT7 level *via* FBXO7-mediated proteolysis.***A*, where indicated, SH-SY5Y cells were treated for 6 h with MPP^+^ (500 μM), 6-OHDA (50 μM), rotenone (10 μM), staurosporine (STS, 0.5 μM), or H_2_O_2_ (500 μM). Cell lysates were immunoblotted with anti-SIRT7 or anti-FBXO7 antibodies. Relative SIRT7 levels were quantified and are presented as the mean ± SD of three independent experiments (∗∗*p* ≤ 0.001; ∗*p* ≤ 0.05; NS, not significant). *B*, SH-SY5Y cells were treated with 500 μM H_2_O_2_ for the indicated times. Cell lysates were immunoblotted with the indicated antibodies. *C*, SH-SY5Y cells were transfected for 48 h with control-siRNA or *FBXO7*-siRNA. The cells were then treated an additional 4 h with 500 μM H_2_O_2_ and immunoblotted with the indicated antibodies. *Asterisks* indicate nonspecific bands. *D*, SH-SY5Y cells were transfected for 24 h with a plasmid encoding FLAG-FBXO7-▵U mutant and treated for an additional 4 h with vehicle (−) or H_2_O_2_ (500 μM). Cell lysates were immunoblotted with the indicated antibodies. Relative levels of SIRT7 were quantified, and the results are presented as the mean ± SD of three independent experiments (∗∗∗*p* ≤ 0.0001; ∗∗*p* ≤ 0.001). *E*, SH-SY5Y cells were transfected for 24 h with plasmids encoding wildtype SIRT7-V5/His or the SIRT7-V5/His-4KR mutant. The cells were then treated an additional 3 h with MG132 (20 μM), vehicle (−) or H_2_O_2_ (500 μM) alone, or in combination, as indicated. Cell lysates were immunoprecipitated using an anti-V5 antibody, followed by immunoblotting with the indicated antibodies. *Asterisks* indicate IgG heavy chains. Tubulin and HSP90 served as loading controls. 6-OHDA, 6-hydroxydopamine; FBXO7, F-box-only protein 7; HSP90, heat shock protein 90; IgG, immunoglobulin G; MPP+, 1-methyl-4-phenylpyridinium; SIRT7, sirtuin 7.

Since several studies have reported that both FBXO7 and SIRT7 act as regulators of the stress response (15, 21, 33, 34, 35), we investigated whether FBXO7-induced SIRT7 degradation affects cell viability in response to various stress inducers. First, SH-SY5Y cells were treated with various neurotoxic stimuli, including MPP^+^, 6-OHDA, rotenone, staurosporine, and H_2_O_2_, which cause neuronal cell death. Treatment with H_2_O_2_ caused a strong reduction in endogenous SIRT7 levels (Fig. 6*A*). H_2_O_2_ is commonly used to cause cellular oxidative stress and damage to cells, which also contributes to the progression of PD. The rate of reduction in endogenous SIRT7 rapidly decreased, which was completed by 4 h after H_2_O_2_ treatment (Fig. 6*B*). In addition, we found that endogenous SIRT7 expression was decreased in a dose-dependent manner by H_2_O_2_ in HEK293 cells (Fig. S4*A*). Next, we examined whether FBXO7 is involved in the H_2_O_2_-induced proteolysis of SIRT7, and whether this requires the catalytic activity of FBXO7. SH-SY5Y cells were transfected with control siRNA or *FBXO7*-siRNA for 48 h, followed by treatment with vehicle or H_2_O_2_. Immunoblotting of cell lysates with anti-SIRT7 antibodies revealed that cells transfected with *FBXO7*-siRNA displayed no significant reduction in SIRT7 levels in response to H_2_O_2_ (Fig. 6*C*). Moreover, when cells overexpressing the FLAG-tagged FBXO7-▵U mutant were treated with H_2_O_2_, they displayed 13-fold higher level of SIRT7 compared with those of cells treated with H_2_O_2_ alone (Fig. 6*D*).

To determine whether FBXO7-mediated polyubiquitination affects SIRT7 proteolysis under H_2_O_2_ treatment, SH-SY5Y cells were transfected for 24 h with plasmids encoding SIRT7-V5/His-WT or SIRT7-V5/His-4KR and then treated with vehicle or H_2_O_2_. As shown in Figure 6*E*, the extent of SIRT7-WT ubiquitination increased in response to H_2_O_2_, whereas this effect was not observed in the polyubiquitination-defective SIRT7-4KR mutant (Fig. 6*E*). Similar to these effects in SH-SY5Y cells, H_2_O_2_ treatment increased SIRT7 ubiquitination in HEK293 cells (Fig. S4*B*). However, the polyubiquitination of SIRT7 under H_2_O_2_ treatment was remarkably inhibited in cells expressing FLAG-FBXO7-▵F, lacking the F-box domain (Fig. S4*B*). These results suggested that H_2_O_2_ treatment triggered FBXO7-mediated SIRT7 ubiquitination and degradation.

### FBXO7-mediated degradation of cytoprotective SIRT7 contributes the H_2_O_2_-induced cell death in SH-SY5Y cells

**Figure 7:**
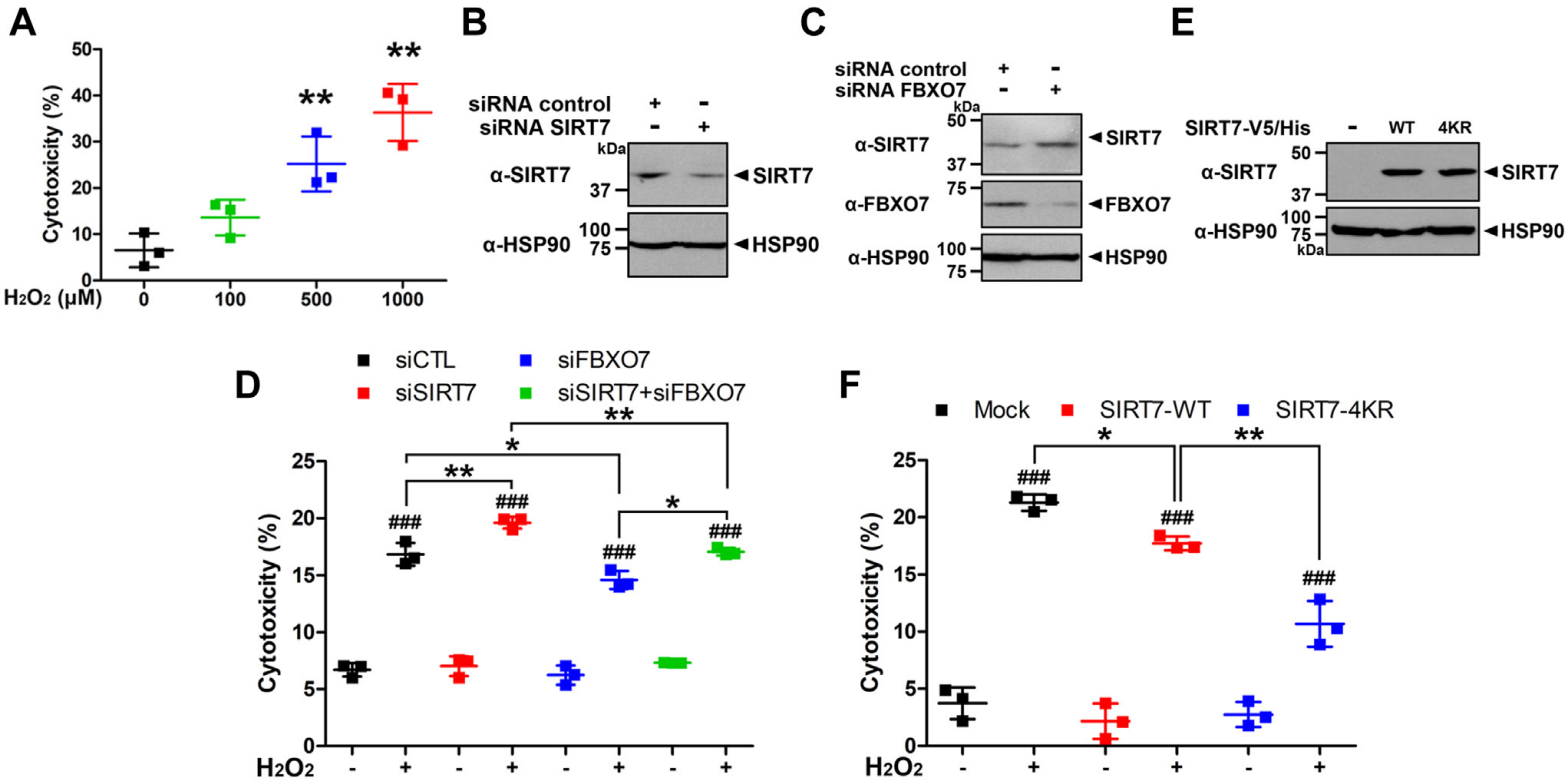
**FBXO7 promotes H**_**2**_**O**_**2**_**-induced cell death *via* the reduction of cytoprotective SIRT7.***A*, SH-SY5Y cells were treated for 6 h with vehicle or the indicated concentrations of H_2_O_2_. Cell toxicity was measured using lactate dehydrogenase (LDH) assays. Data are presented as the mean ± SD of three independent experiments (∗∗*p* ≤ 0.001). *B*, SH-SY5Y cells were transfected for 48 h with control-siRNA or *SIRT7*-siRNA. Cell lysates were immunoblotted with the indicated antibodies. *C*, SH-SY5Y cells were transfected for 48 h with control-siRNA or *FBXO7*-siRNA, and the cell lysates were immunoblotted with anti-SIRT7 or anti-FBXO7 antibodies. *D*, SH-SY5Y cells were transfected for 48 h with control-, *SIRT7*-, or *FBXO7-*siRNA and then treated for an additional 6 h with vehicle or H_2_O_2_ (500 μM). Data are presented as the mean ± SD of three independent experiments. *Asterisks* refer to statistically significant differences between transfection, which were analyzed by one-way ANOVA followed by Turkey’s post-test (∗∗*p* ≤ 0.001; ∗*p* ≤ 0). Sharp indicated significant differences between control and treatments, which were analyzed by two-way ANOVA followed by Turkey’s post-test (^###^*p* ≤ 0.0001). *E*, SH-SY5Y cells were transfected for 24 h with plasmids encoding wildtype SIRT7-V5/His or SIRT7-V5/His-4KR mutant, and the cell lysates were immunoblotted with anti-V5 antibodies. HSP90 served as a loading control. *F*, SH-SY5Y cells were transfected for 24 h with plasmids encoding wildtype SIRT7-V5/His or the SIRT7-V5/His-4KR mutant and then treated for an additional 6 h with vehicle or H_2_O_2_ (500 μM). Data are presented as the mean ± SD of three independent experiments. *Asterisks* refer to statistically significant differences between transfection, which were analyzed by one-way ANOVA followed by Turkey’s post-test (∗∗*p* ≤ 0.001; ∗*p* ≤ 0). Sharp indicated significant differences between control and treatments, which were analyzed by two-way ANOVA followed by Turkey’s post-test (^###^*p* ≤ 0.0001). FBXO7, F-box-only protein 7; H_2_O_2_, hydrogen peroxide; HSP90, heat shock protein 90; SIRT7, sirtuin 7.

Several reports have demonstrated that SIRT7 has cytoprotective functions against toxic oxidative stressors (33, 34, 35). Based on these findings, we explored whether FBXO7-mediated SIRT7 degradation affects cell viability or cytotoxicity under H_2_O_2_ treatment. First, SH-SY5Y cells were treated with various concentrations of H_2_O_2_, and the change in cell viability was measured using the lactate dehydrogenase (LDH) cytotoxicity assay. As shown in Figure 7*A*, the cytotoxicity of H_2_O_2_ increased in a dose-dependent manner. Next, we assessed the knockdown effect of *FBXO7* or *SIRT7* gene expression on H_2_O_2_-induced SH-SY5Y cell death. Immunoblotting analysis revealed that the expression of endogenous FBXO7 and SIRT7 was efficiently downregulated by *FBXO7*-siRNA and *SIRT7*-siRNA, respectively, compared with that of the negative control–transfected group, confirming the transfection efficiency. In addition, endogenous SIRT7 levels were increased by transfection of *FBXO7*-siRNA (Fig. 7, *B* and *C*). Consistent with the findings of previous reports, siRNA-mediated *SIRT7* knockdown alone exhibited a 2.8% higher level of H_2_O_2_-induced cytotoxicity compared with that of cells transfected with control siRNA. In contrast, cells with siRNA-mediated *FBXO7* knockdown alone showed a 2.3% lower level of H_2_O_2_-induced cytotoxicity compared with that of cells transfected with control siRNA. Furthermore, cells transfected with both *SIRT7*- and *FBXO7*-siRNA followed by treatment with H_2_O_2_ displayed much higher cytotoxicity compared with that of cells transfected with *FBXO7*-siRNA alone (Fig. 7*D*).

We further examined whether H_2_O_2_ treatment caused a significant decrease in SH-SY5Y cell viability in the presence of the SIRT7-4KR mutant. As a control, we examined the transfection efficiency of SIRT7-WT and SIRT7-4KR in SH-SY5Y cells (Fig. 7*E*). The cells were mock transfected or transfected for 24 h with plasmids encoding SIRT7-V5/His-WT or SIRT7-V5/His-4KR mutant and in addition treated with vehicle or H_2_O_2_. Consistent with the results shown in Figure 7*D*, cells overexpressing exogenous SIRT7-WT showed a 3.6% lower level of H_2_O_2_-induced cytotoxicity compared with that of mock-transfected cells. Interestingly, cells overexpressing the exogenous SIRT7-4KR mutant and treated with H_2_O_2_ displayed a 7% lower level of cytotoxicity than that of cells expressing SIRT7-WT alone (Fig. 7*F*). These results are identical to those shown in Figure 5*D* and suggest that the four lysine residues of SIRT7 and their polyubiquitination are important for H_2_O_2_-induced cell death in SH-SY5Y cells.

Taken together, these results suggest that FBXO7 contributes to H_2_O_2_-induced neuronal cell death by reducing cytoprotective SIRT7 levels.

### The PD-linked FBXO7-R498X mutant does not alter the stability of SIRT7

**Figure 8:**
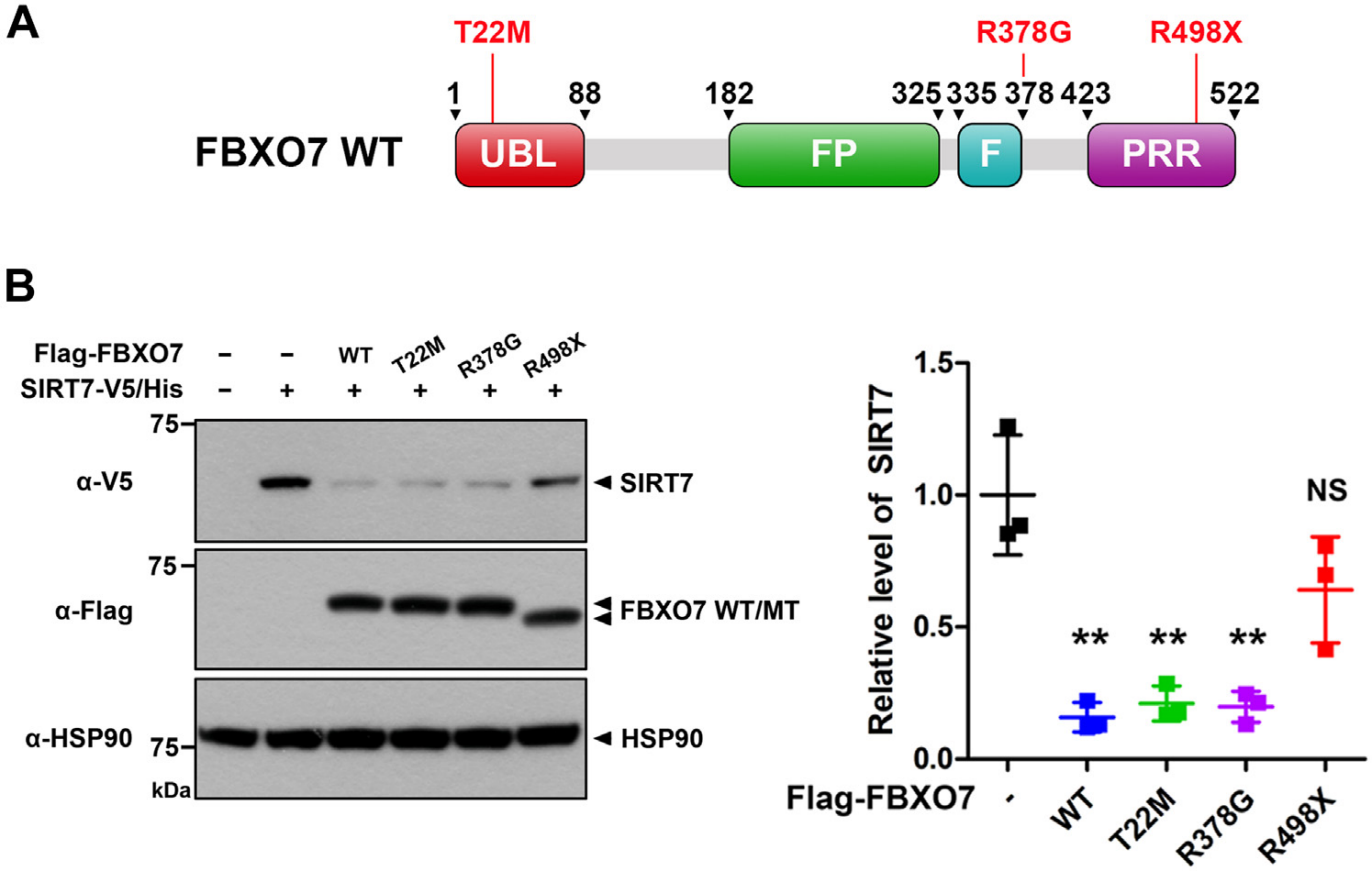
**The Parkinson’s disease (PD)–linked FBXO7-R498X mutant does not alter the stability of SIRT7.***A*, schematic of PD-linked mutations in FBXO7. *B*, where specified, HEK293 cells were transfected for 24 h with plasmids encoding SIRT7-V5/His, wildtype FLAG-FBXO7, FLAG-FBXO7-T22M, FLAG-FBXO7-R378G, or FLAG-FBXO7-R498X mutant alone or in combination. Cell lysates were immunoblotted with the indicated antibodies. Relative levels of SIRT7 were quantified, and the results are represented as the mean ± SD of three independent experiments (∗∗*p* ≤ 0.001; NS, not significant). HSP90 served as a loading control. HEK293, human embryonic kidney 293 cell line; HSP90, heat shock protein 90; SIRT7, sirtuin 7.

Finally, we investigated the effect of three common familial PD-linked pathogenic mutants of FBXO7 (FBXO7-T22M, FBXO7-R378G, and FBXO7-R498X; Fig. 8*A*) on the degradation of SIRT7. The T22M mutation of FBXO7 in the N-terminal Ubl domain suppresses mitophagy by preventing its interaction with parkin, the R378G mutation is located at the end of the F-box domain and reduces the affinity of FBXO7 for Skp1 in the SCF complex, and the nonsense R498X mutation results in the deletion of C-terminal 24 amino acids from the proline-rich region domain, suppressing the E3 ligase activity of FBXO7 (10, 11). Cells were transfected with SIRT7 alone or in combination with FBXO7-WT or one of these FBXO7 mutants. As shown in Figure 8*B*, FBXO7-T22M and FBXO7-R378G mutants caused a reduction in SIRT7 levels, similar to that of FBXO7-WT. However, the FBXO7-R498X mutant had no effect on the stability of SIRT7 (Fig. 8*B*). These results are entirely consistent with the finding that FBXO7 facilitates the degradation of SIRT7 in an SCF-dependent manner, and the catalytic activity of FBXO7 was efficiently suppressed by the R498X substitution.

Collectively, these results indicated that SCF^FBXO7^ E3 ligase activity plays an essential role in the regulation of SIRT7 stability. Moreover, PD patients with mutations in FBXO7-R498X might have defects in the regulation of SIRT7-linked various cellular functions.

## Discussion

Several reports have strongly indicated a close relationship between the mammalian sirtuin family and the pathogenesis of neurodegenerative diseases, including PD. Among the primary hallmarks of PD, including toxic protein aggregation, mitochondria dysfunction, and neuronal cell death, aging is also regarded as a crucial factor (36). Although sirtuins act as NAD^+^-dependent protein deacetylases that consume NAD^+^, NAD^+^ levels decrease with age in many species, including humans (37, 38). According to a previous report, NAD^+^ deficiency aggravates the inactivation of SIRT1 in the nucleus and mitochondria, causing the impairment of mitophagy and leading to neurodegeneration (39). In addition, SIRT3 and SIRT5 have shown neuroprotective effects in PD models, whereas SIRT2 and SIRT6 were found to enhance PD pathology (29, 40, 41, 42). These reports suggest that understanding the precise regulatory mechanism of sirtuins could provide valuable insights for the development of novel and efficient PD therapeutics. Nonetheless, the functional role of SIRT7 in PD has not been elucidated. Here, we provided the first evidence of a close relationship between SIRT7 and PD pathogenesis. The current study demonstrated that PD-associated FBXO7 promotes the ubiquitination and degradation of SIRT7 in mammalian cells, reducing the cytoprotective effect of SIRT7 during H_2_O_2_-induced SH-SY5Y cell death.

FBXO7, a component of the SCF ubiquitin E3 ligase complex, has dual functions, including SCF-dependent canonical and SCF-independent noncanonical activity (8). As an example of its noncanonical function, we previously reported that FBXO7 facilitates the proteolysis of aging-related FOXO4 *via* caspase-8 activation in mammalian cells (15). This finding was further supported by reports that Nutcracker, the *Drosophila* ortholog of FBXO7, activates caspase, stimulating the proteasome during sperm differentiation in *Drosophila* (43, 44). In contrast, the capacity of FBXO7 to function as an E3 ligase has been described for several substrate proteins involved in UPS degradation (8). Our study presents additional evidence for the canonical role of FBXO7 in the latter case, along with identifying SIRT7 as its novel target. In addition to FBXO7, SIRT7 has also been reported to be polyubiquitinated by HIV-1 Vpr (45), a multifunctional accessory protein critical for efficient viral infection of target cells. Vpr interacts with the CRL4–DCAF1 ubiquitin E3 ligase complex and induces the proteasome degradation of SIRT7 through CRL4–DCAF1 (45). Similar to the FBXO7-ΔU mutant lacking the Ubl domain, and thus disabling the recruitment of substrates for ubiquitination, the PD-linked FBXO7-R498X mutation, which is known to inhibit SCF^FBXO7^ E3 ligase activity, did not reduce SIRT7 stability. These results demonstrate that FBXO7 promotes the degradation of SIRT7 through the SCF^FBXO7^-dependent UPS pathway.

Polyubiquitination involves the sequential attachment of ubiquitin molecules to lysine residues on the target substrates or on itself (46). The ubiquitin molecule can be ubiquitinated at its eight residues (M1, K6, K11, K27, K29, K33, K48, and K63) (47). Among these sites, K48- and K63-linked ubiquitin chains have been well characterized. K48-linked polyubiquitination mainly targets substrates for proteasome, whereas K63-linked monoubiquitinaton or polyubiquitination is commonly used in various cellular responses and signaling pathways such as DNA damage, kinase activation, and NF-κB activation, without disintegrating the substrates (47). In addition to the proteasome system, some ubiquitinated substrates can be degraded *via* the lysosomal autophagy pathway (48). In the present study, we found that FBXO7 promoted the K48-linked polyubiquitination of SIRT7. In particular, we found that this occurred at four lysine residues (K292, K395, K397, and K398) of SIRT7. When these four SIRT7 residues were mutated to arginine, FBXO7-mediated degradation and ubiquitination were markedly blocked, as expected. Interestingly, the SIRT7-4KR mutant exhibited higher deacetylase activity than that of SIRT7-WT, displaying much stronger repression of downstream *RPS20* mRNA expression. This action could be observed from two aspects: On the one hand, compared with the samples expressing SIRT-WT, which could be degraded by endogenous FBXO7, the SIRT7-4KR mutant could be resistant to degradation, and thus the cells should have much larger wildtype-like SIRT7 levels and display much higher activity. On the other hand, mutations of these four lysine sites could affect the protein conformation, facilitating much higher catalytic activity.

In addition to the modulatory role in aging and longevity, sirtuins, including SIRT7, have also been associated with several oxidative stress–related cellular reactions, such as the DNA repair mechanism, mitochondrial function, and metabolic pathways (49). For example, cardiomyocytes from *Sirt7*-knockout mice showed increased numbers of apoptotic cells in response to H_2_O_2_ (33). In addition, endogenous SIRT7 levels in embryonal rat heart–derived H9c9 cells were slightly decreased under H_2_O_2_-induced oxidative stress. Treatment with resveratrol, a chemical activator of SIRT1, 3, 4, and 7, rescued H_2_O_2_-induced SIRT7 reduction and cytotoxicity (34). A recent study found that H_2_O_2_ downregulated the expression of SIRT7 and upregulated the expression of miR-335-5p in human umbilical vein endothelial cells (35). siRNA-mediated *SIRT7* knockdown induced premature endothelial senescence and decreased the proliferation, adhesion, migration, and nitric oxide secretion in human umbilical vein endothelial cells. Moreover, the miR-335-5p inhibitor attenuated the downregulated SIRT7 expression induced by oxidative stress, and SIRT7 overexpression rescued miR-335-5p-induced endothelial dysfunction. Overall, our results and these previous findings suggest that H_2_O_2_ decreases the cytoprotective effect of SIRT7 in mammalian cells (35).

FBXO7 can exhibit either cellular protective or cytotoxic activity in response to various stresses depending on the cellular context. Regarding the cytoprotective effect, FBXO7 interacts with parkin and is involved in the recruitment of parkin into impaired mitochondria to promote mitophagy (14). Under mild and transient stress, FBXO7 expression is upregulated, which facilitates mitophagy to protect the cells (50). Alternatively, FBXO7 exerts cytotoxic activity. For example, FBXO7 promotes PINK1 ubiquitination and degradation. As PINK1 has a cytoprotective effect, treatment with an FBXO7 inhibitor could improve cell viability and reduce dendrite injury against MPP^+^ toxicity in human SH-SY5Y cells and primary neurons (21). Furthermore, we previously showed that 6-OHDA treatment triggers the FBXO7-mediated proteolysis of FOXO4 *via* caspase-8 activation in mouse MN9D cells (15). Specifically, we demonstrated that FBXO7 augmented the cytotoxicity of 6-OHDA and attenuates the cytoprotective effects of FOXO4 in MN9D cells. The current study provides additional evidence that FBXO7 displays neurotoxic action, which may be produced in part *via* SIRT7 degradation. Furthermore, FBXO7 aggravated SH-SY5Y cell death in response to H_2_O_2_.

In conclusion, the current study proposes FBXO7 as a novel mediator for the ubiquitination of SIRT7. These findings also suggest that the FBXO7-mediated downregulation of cytoprotective SIRT7 may cause or be closely associated with cell death and might even contribute to the pathogenesis of PD.

## Experimental procedures

### Materials

Dulbecco's modified Eagle's medium (DMEM), fetal bovine serum (FBS), Lipofectamine 2000, polyethylenimine reagent, and antimouse and anti-rabbit horseradish peroxidase (HRP)–conjugated secondary antibodies were purchased from PerkinElmer and Analytical Sciences. *Trans*IT-X2 Dynamic Delivery System for plasmid DNA and siRNA transfection was purchased from Mirus Bio. Protein A Sepharose beads were purchased from GE Healthcare Biosciences. The proteasome inhibitor MG132 and epoxomicin were purchased from A.G. Scientific and Millipore, respectively. NH_4_Cl and cycloheximide were purchased from Sigma–Aldrich. MLN4924, a chemical inhibitor of NEDD8-activating enzyme, was purchased from Selleck Chemicals. Enhanced chemiluminescence reagent was purchased from AbClon and Advansta. All other chemicals used in the study were analytical-grade commercial products purchased from Sigma–Aldrich. The following primary antibodies (and their catalog numbers) for Western blotting were purchased from the indicated vendors: mouse monoclonal anti-HA (sc-7392; Santa Cruz Biotechnology), anti-FLAG (F3165; Sigma–Aldrich), anti-Myc (sc-40; Santa Cruz Biotechnology), anti-V5 (R960-25; Thermo Fisher Scientific), anti-FBXO7 (sc-271763; Santa Cruz Biotechnology), anti-SIRT7 (sc-365344; Santa Cruz Biotechnology), antiubiquitin (Ubi; sc-8017; Santa Cruz Biotechnology), anti-K-63 Ubi (05-1313; Millipore), anti–heat shock protein 90 (sc-13119; Santa Cruz Biotechnology), rabbit monoclonal anti-SIRT7 (5360; Cell Signaling Technology), anti-K-48 Ubi (05-1307; Millipore), anti-H3K18ac (ab1191; Abcam), antihistone 3 (ab1791; Abcam), H4K5ac (07-327; Millipore), antihistone 4 (07-108; Millipore), and anti-FBXO7 (sc-86450; Santa Cruz Biotechnology). HRP-conjugated antimouse (AP124P) and anti-rabbit (AP132P) secondary antibodies were purchased from EMD Millipore.

### DNA constructs and RNA interference

Mammalian expression vectors for FLAG-tagged human wildtype FBXO7 isoform-1 and its F-box deleting mutant were provided by Dr H.J. Kuiken (The Netherlands Cancer Institute). The mammalian expression vector for V5/His-tagged human wildtype SIRT7 was provided by Dr K.Y. Choi (Pohang University of Science and Technology). Plasmids encoding FLAG-tagged SIRT2, SIRT3, SIRT4, and SIRT7 were kindly provided by H.S. Kim (Ewha Womans University). Plasmids encoding FLAG-tagged SIRT1, SIRT5, and SIRT6 were provided by Y.J. Oh (Yonsei University). All plasmid sequences were verified using DNA sequencing (Bionics). siRNAs for *FBXO7*, *SIRT7*, and *CUL1*, and control scrambled siRNA (catalog no.: 51-01-14-04) were designed and synthesized by Integrated DNA Technologies (Hanam-si). *FBXO7*-specific siRNA duplex sequences were 5′-UUGGUUCUCCUCUAGAUUGAAGU(dTdT)-3′ (sense) and 5′-ACUUCAAUCUAGAGGAGAACCAA(dTdT)-3′ (antisense). *SIRT7*-specific siRNA duplex sequences were 5′-GUGUGAACUUUAUAGAAU(dTdT)-3′ (sense) and 5′-AGAGAGGAUUCUAUAAAG(dTdT)-3′ (antisense). The *CUL1*-specific siRNA duplex sequences were 5′-CAGGUUUACCUUCAUGAAAGCACAC(dTdT)-3′ (sense) and 5′-GUGUGCUUUCAUGAAGGUAAACCUGAA(dTdT)-3′ (antisense).

### Cell culture and DNA transfection

HEK293 cells were maintained in DMEM supplemented with 10% FBS and 100 U/ml penicillin–streptomycin. *FBXO7*-knockout HAP1 cells were purchased from Horizon Discovery (catalog no.: HZGHC002684c003) and maintained in Iscove’s modified Dulbecco’s medium supplemented with 10% FBS and 100 U/ml penicillin–streptomycin. Human neuroblastoma SH-SY5Y cells were maintained in DMEM/F12 supplemented with 10% FBS and 100 U/ml penicillin–streptomycin. All cells were grown at 37 °C with 5% CO_2,_ unless otherwise indicated. All DNA transfections were carried out using polyethylenimine, Lipofectamine 2000, or Mirus reagent, according to the manufacturer’s instructions.

### Coimmunoprecipitation and immunoblot analysis

Cell lysates were washed with ice-cold PBS, scraped, and mixed with 1% Nonidet P-40 lysis buffer (50 mM Tris, pH 7.5; 1% Nonidet P-40, 150 mM NaCl, 10% glycerol, 1 mM sodium orthovanadate, 1 μg/ml aprotinin, 1 mM EGTA, 1 mM sodium fluoride, and 0.2 mM phenylmethylsulfonyl fluoride). The cells were sonicated, and the supernatants were collected by centrifugation at 13,000*g* for 15 min at 4 °C. For immunoprecipitation, 500 to 1000 μg of cell lysates were incubated with 1 μg of appropriate antibodies overnight at 4 °C with gentle rotation. Protein A Sepharose beads were then added and incubated for 2 h at 4 °C with gentle rotation. The beads were washed with 1% Nonidet P-40 lysis buffer, and the immunocomplexes were dissociated by boiling in 2× sample buffer. The samples were then resolved by SDS-polyacrylamide gel electrophoresis and transferred to a nitrocellulose membrane (Whatman, GE Healthcare Life Sciences). Membranes were blocked in TBST buffer (20 mM Tris, pH 7.5, 137 mM NaCl, and 0.1% Tween-20) and 5% nonfat dry milk for 1 h at room temperature. The membranes were then incubated with the appropriate primary antibody at 4 °C overnight. Membranes were washed in TBST and incubated with HRP-conjugated secondary antibody for 2 h at room temperature. The membranes were washed with TBST again, and the protein bands were visualized using Enhanced chemiluminescence reagents, according to the manufacturer’s instructions.

### Preparation of mouse whole-brain lysates

Whole brains were obtained from 6-week-old male C57BL/6 mice (Orient). Brain tissues were homogenized and sonicated in lysis buffer containing 50 mM Tris (pH 7.4), 150 mM NaCl, 1% Triton X-100, 0.5% sodium deoxycholate, 0.1% SDS, and a protease inhibitor cocktail (Sigma–Aldrich). The samples were centrifuged at 13,000*g* for 20 min at 4 °C, and the supernatants were collected.

### Immunocytochemistry analysis

Human SH-SY5Y cells were seeded onto poly-d-lysine–coated cover glasses. Adherent cells were washed twice with PBS and immediately fixed in 3.7% formaldehyde for 10 min at room temperature. After fixation, the cells were permeabilized with 0.1% Triton X-100 for 10 min and blocked with 1% bovine serum albumin in TBST for 1 h at room temperature. The cells were immunostained using rabbit polyclonal anti-SIRT7 and/or mouse monoclonal anti-FBXO7 antibodies, washed, and incubated with Alexa Fluor 488– or Alexa Fluor 594–conjugated anti-immunoglobulin G antibodies. Images were captured using an LSM 880 confocal microscope (Carl Zeiss) and processed using the Zeiss LSM Image Browser (Carl Zeiss).

### RNA extraction and real-time analysis

Total RNA was isolated from the cells, and target mRNAs were amplified by real-time PCR using complementary DNA as the template. The primer sequences for *RPS20* and *GAPDH* were as follows: *RPS20*—forward: 5′-AGGGCTGAGGATTTTTGGTC-3′; *RPS20*—reverse: 5′-GGGTGTTTTTCCGGTATCCT-3′, *GAPDH*—forward: 5′-TGCACCACCAACTGCTTAGC-3′, and *GAPDH*—reverse: 5′-GGCATGGACTGTGGTCATGAG-3′.

### LDH cytotoxicity assays

Cytotoxicity was evaluated using an LDH Cytotoxic Detection Kit (Takara). SH-SY5Y cells were transfected with either siRNA for 48 h or DNA for 24 h, followed by treatment with 500 μM H_2_O_2_ for an additional 6 h. Cell-free culture media were collected and used in the LDH assay, according to the manufacturer’s instructions. The maximum LDH release (referred to as “high control”) was determined by solubilizing the cells in 1% Triton X-100; the spontaneous LDH release (referred to as “low control”) was determined by incubating the cells in medium alone. The absorbance was measured at 490 nm using a microplate reader. The cytotoxicity was calculated as a percentage of the control using the formula: cytotoxicity = [(experimental value − low control)/(high control − low control)] × 100%.

### Statistical analysis

Unpaired Student’s *t* tests were used for statistical analyses to compare data from different groups. Cell cytotoxicity were analyzed using the one-way ANOVA and two-way ANOVA followed by Tukey’s post-test. The analysis was performed using GraphPad Prism software (version 5; GraphPad Software, Inc). All values are reported as mean ± SD of at least three independent experiments. The intensities of the Western blot bands were measured using GelQuant.NET software (version 1.8.2; biochemlabsolutions.com).

## Data availability

All datasets are included within the article or are available from the corresponding author: Kwang Chul Chung (kchung@yonsei.ac.kr).

## Supporting information

This article contains supporting information.

## Conflict of interest

The authors declare that they have no conflicts of interest with the contents of this article.
