# Supplementary material for: E3 ligase adaptor FBXO7 contributes to ubiquitination and proteasomal degradation of SIRT7 and promotes cell death in response to hydrogen peroxide
Source: J Biol Chem. 2023 Jan 13;299(3):102909. doi: 10.1016/j.jbc.2023.102909 (PMC9971319; doi:10.1016/j.jbc.2023.102909)
Supplement: Supplemental Figures S1–S4 [file mmc1.pdf]

Supplementary data

E3 ligase adaptor FBXO7 contributes to ubiquitination and proteasomal degradation of SIRT7 and promotes cell death in response to hydrogen peroxide

Su Hyoun Lee, Yun Ju Lee, Sungyeon Jung, and Kwang Chul Chung

Content: Supporting Figures and Legends

1. Figure S1
2. Figure S2
3. Figure S3
4. Figure S4

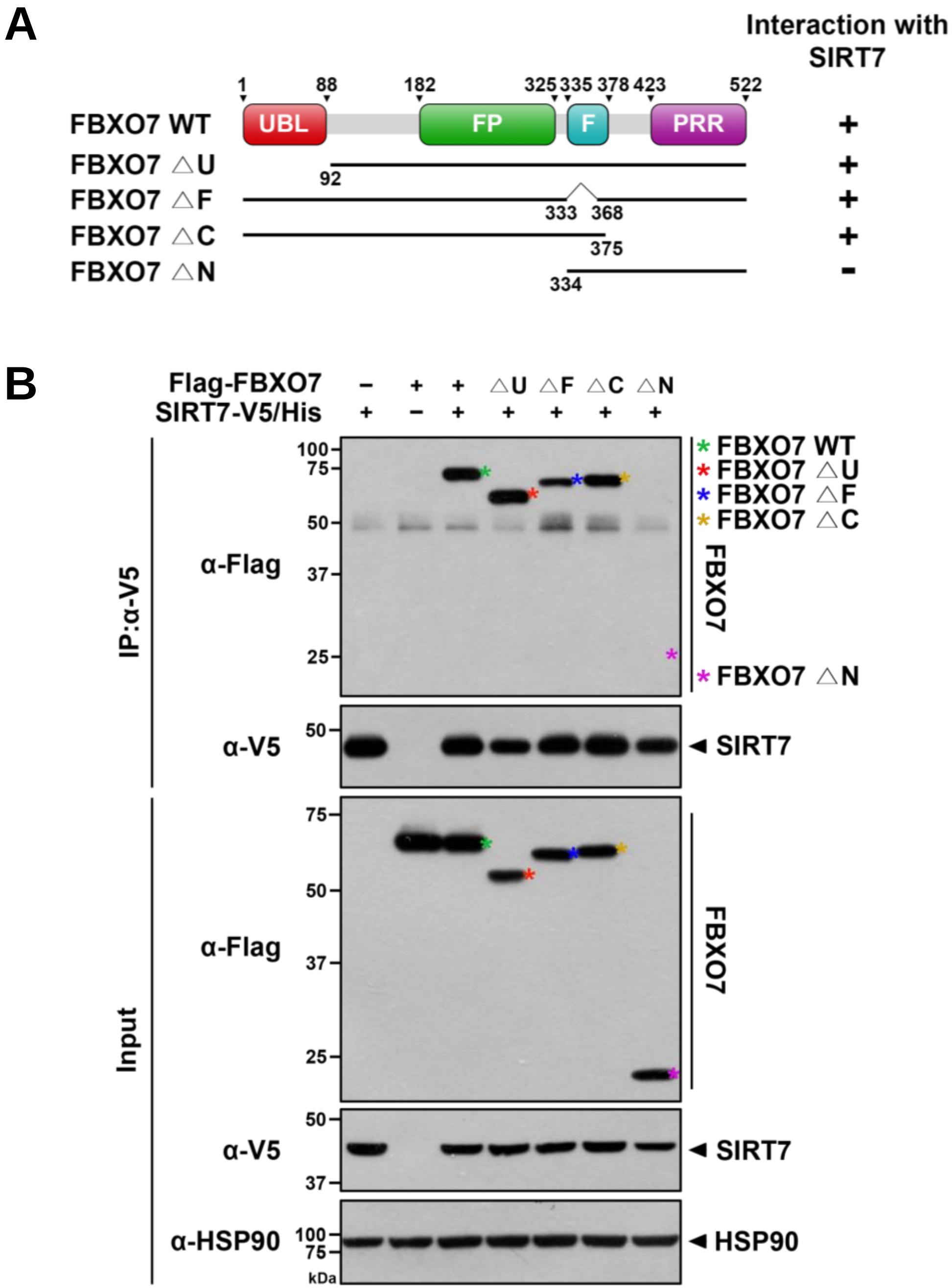
A
Interaction with SIRT7
FBXO7 WT
UBL
FP
F
PRR
1
88
182
325
335
378
423
522
FBXO7 △U
92
FBXO7 △F
333
368
FBXO7 △C
375
FBXO7 △N
334
+
+
+
+
-
B
Flag-FBXO7
SIRT7-V5/His
IP:α-V5
Input
α-Flag
α-V5
α-HSP90
100
75
50
37
25
kDa
FBXO7 WT
FBXO7 △U
FBXO7 △F
FBXO7 △C
FBXO7 △N
FBXO7
SIRT7
HSP90

Figure S1. The region spanning amino acid residues 92-333 of FBXO7, including the FP domain, is important for SIRT7 binding. *A*, Schematic of wild-type FBXO7 (FBXO7-WT) and its deletion mutants. The results from co-immunoprecipitation and binding assays between SIRT7 and either FBXO7-WT or one of its deletion mutants are shown on the right side. Minus (-) indicates no binding and plus (+) indicates binding. *B*, Where indicated, HEK293 cells were transfected for 24 h with plasmids encoding SIRT7-V5/His, Flag-tagged wild-type FBXO7, or one of its deletion mutants alone or in combination. Cell lysates were immunoprecipitated using anti-V5 antibody, followed by immunoblotting with the indicated antibodies. HSP90 served as a loading control.

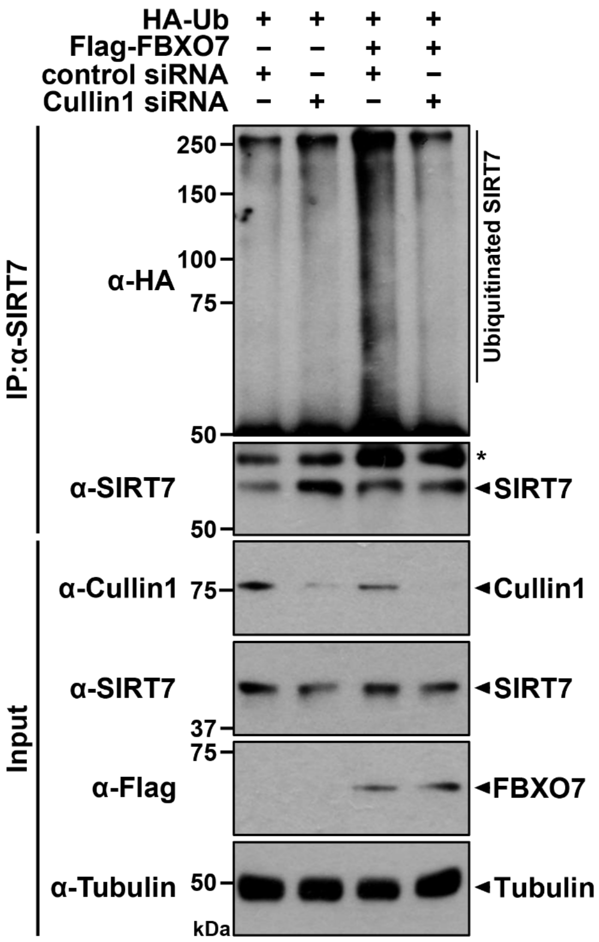

Figure S2. FBXO7-mediated SIRT7 polyubiquitination is dependent on the SCF complex. Where specified, HEK293 cells were transfected for 48 h with control-siRNA, *CUL1*-siRNA, or the plasmids encoding HA-ubiquitin (Ub) or Flag-FBXO7 alone or in combination, and then treated for 6 h with 20 μM MG132. Cell lysates were immunoprecipitated using an anti-SIRT7 antibody, and the precipitates were immunoblotted with the indicated antibodies. Asterisks indicate IgG heavy chains. Tubulin served as a loading control.

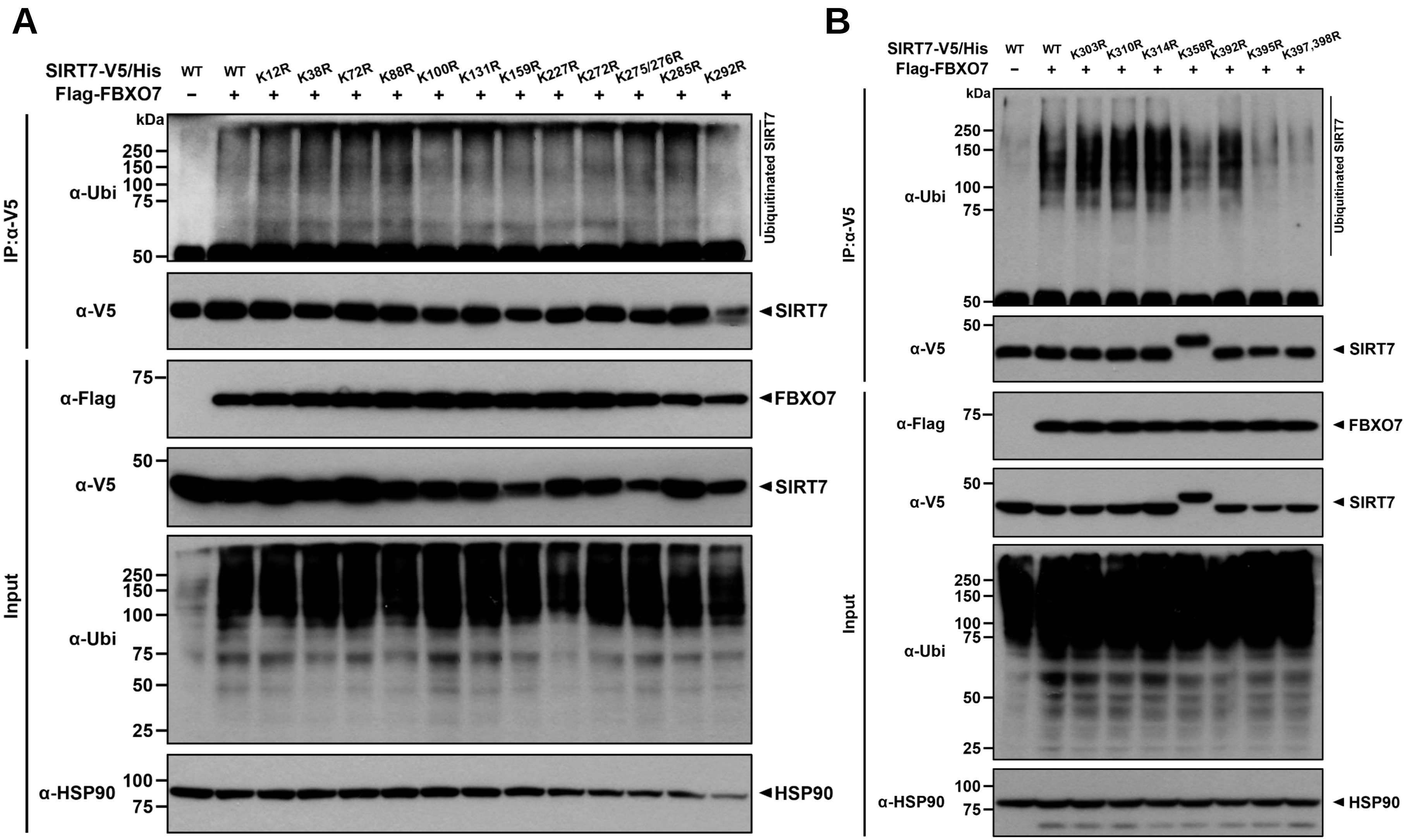
A
SIRT7-V5/His
Flag-FBXO7
WT WT K12R K38R K72R K88R K100R K131R K159R K227R K272R K275/276R K285R K292R
IP:α-V5
Input
α-Ubi
α-V5
α-Flag
α-HSP90
kDa
Ubiquitinated SIRT7
SIRT7
FBXO7
HSP90
B
SIRT7-V5/His
Flag-FBXO7
WT WT K303R K310R K314R K358R K392R K395R K397,398R

Figure S3. Mapping of the polyubiquitination sites on SIRT7 by FBXO7. *A* and *B*, Where indicated, HEK293 cells were transfected for 24 h with plasmids encoding SIRT7-V5/His-WT, one of its point-mutants, and/or Flag-FBXO7, and then treated for an additional 6 h with 20 μM MG132. Cell lysates were immunoprecipitated using anti-V5 antibody, followed by immunoblotting with the indicated antibodies. HSP90 served as a loading control.

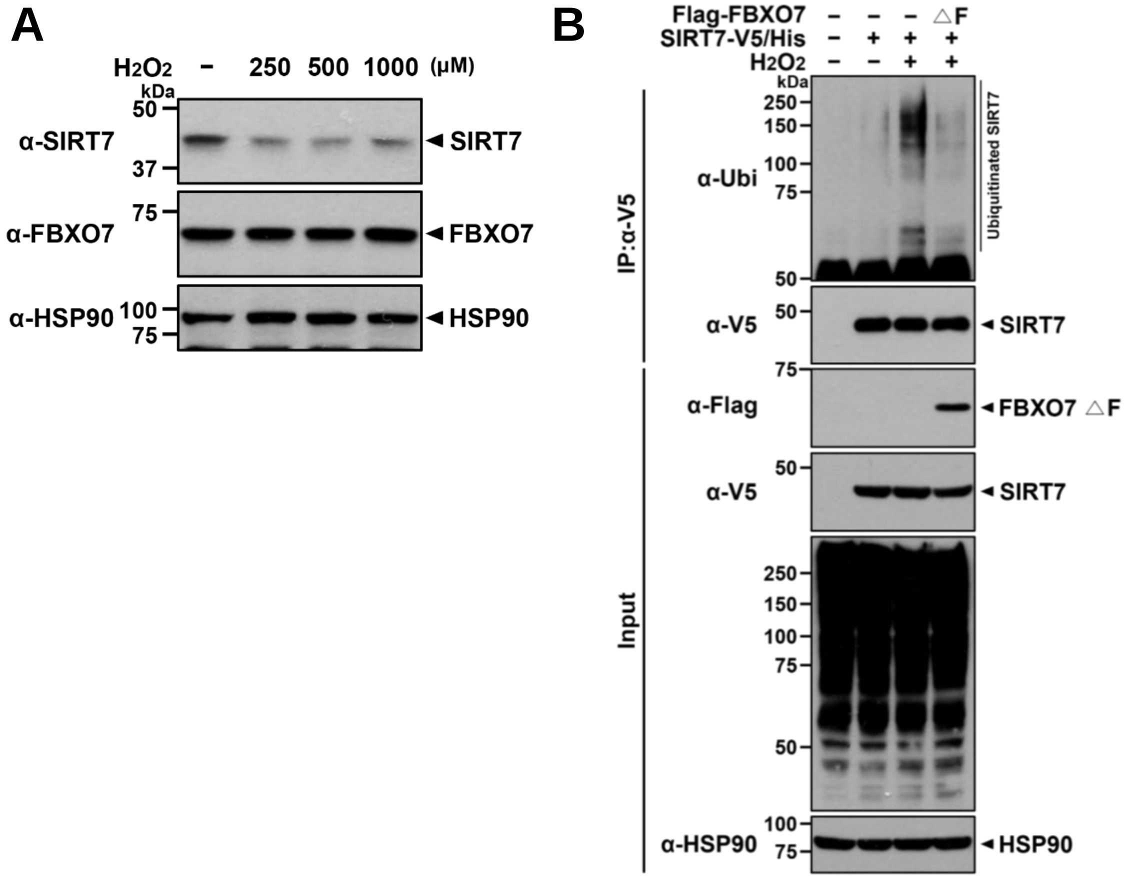

Figure S4. FBXO7 mediated the polyubiquitination of SIRT7 under the treatment of H_2O_2 in HEK293 cells. *A*, HEK293 cells were treated for 12 h with vehicle or the indicated concentrations of H_2O_2. Cell lysates were immunoblotted with anti-SIRT7 or anti-FBXO7 antibodies. *B*, HEK293 cells were transfected for 24 h with the plasmids encoding SIRT7-V5/His and/or Flag-FBXO7-△F, and then treated for 12 h with 500 μM H_2O_2 and for an additional 6 h with 20 μM MG132. Cell lysates were immunoprecipitated using an anti-V5 antibody and the precipitates were immunoblotted with the indicated antibodies. HSP90 served as a loading control.
